# Exposure to workplace bullying and workplace violence/threats of violence and subsequent working life expectancy among older workers: A Swedish prospective cohort study

**DOI:** 10.5271/sjweh.4320

**Published:** 2026-09-01

**Authors:** Rebecka Holmgren, Holendro Singh Chungkham, Linda Magnusson Hanson, Tianwei Xu, Kristina Alexanderson, Kristin Farrants, Martin Hyde, Robin S Högnäs, Reiner Rugulies, Hugo Westerlund

**Affiliations:** 1 Division of Psychobiology and Epidemiology, Department of Psychology, Stockholm University, Stockholm, Sweden.; 2 Department of Public Health Sciences, Stockholm University, Stockholm, Sweden.; 3 Department of Clinical Neuroscience, Karolinska Institutet, Stockholm, Sweden.; 4 School of Business, University of Leicester, Leicester, United Kingdom.; 5 National Research Centre for the Working Environment, Lersø Parkallé 105, DK-2100 Copenhagen, Denmark.; 6 Section of Epidemiology, Department of Public Health, University of Copenhagen, Copenhagen, Denmark.

**Keywords:** labor market participation, occupational health, psychosocial working condition

## Abstract

**Objectives:**

This study aimed to quantify possible associations between exposure to workplace bullying and violence, or threats thereof, and working life expectancy (WLE) among older workers in Sweden.

**Methods:**

We conducted a prospective cohort study using data on 12 920 individuals in analyses of workplace bullying and 12 903 individuals in analyses of violence. Participants, aged ≥50 at baseline, were obtained from the Swedish Longitudinal Occupational Survey of Health, using data from 2008 to 2020. We applied continuous-time multistate Markov models to estimate the average number of years that individuals could be expected to work beyond the age of 50, comparing those exposed to workplace bullying or workplace violence/threats of violence to those unexposed.

**Results:**

In the unadjusted analysis, exposure to workplace bullying and workplace violence/threats of violence was associated with shorter WLE among older workers [bullying: -0.42 years, 95% confidence interval (CI) -1.23–0.01; violence: -0.42 years, 95% CI -1.06–0.01]. After controlling for sex and occupational level, WLE at age 50 was -0.56 years among those exposed to workplace violence (95% CI -1.02– -0.10) compared with the unexposed. The estimate for workplace bullying was -0.63 years (95% CI -1.39–0.14).

**Conclusion:**

Exposure to workplace violence or threats of violence was associated with fewer average expected years in work from age 50. This suggests that prevention of workplace violence could contribute to extended working lives. The association between workplace bullying and WLE was of similar magnitude but not statistically significant.

As life expectancy rises, the number and proportion of older individuals are also increasing (1, 2). To address challenges and realize the opportunities posed by this demographic shift, several policy changes have been introduced around the world, including raising the statutory retirement age to keep older workers in the labor market (3). Thus, from a labor market policy perspective, knowledge about the conditions that promote or hinder extended work participation becomes crucial. The psychosocial work environment, defined as how jobs are designed and how work is organized, managed, and governed (4), may be an important arena for understanding labor market participation among older workers. Adverse psychosocial working conditions, including workplace offensive behaviors, can negatively influence worker’s health, capacity and motivation to remain in the labor market (5–7) but may also be modifiable and amenable to preventive interventions (6, 8).

Prior research has suggested that favorable working conditions contribute to longer working lives. For example, a recent Danish cohort study reported that favorable psychosocial working conditions, such as having influence and receiving recognition at work, were linked to working beyond the state pension age (9). Conversely, studies show that adverse working conditions may increase the risk of early labor market exits, while also reducing remaining years of healthy life. A study, using data from 1.6 million workers in Denmark, reported that higher physical workload was associated with shorter working life expectancy (WLE) among both women and men (10). A UK cohort study of workers aged ≥50, reported that lack of support and low decision authority, respectively, were associated with 1.7 and 1.8 fewer years of healthy WLE (11). Similarly, a Swedish cohort study reported that older workers who experienced job strain at age 50 worked an average of 6–12 fewer months than those who did not (12). However, we are not aware of any studies to date which have examined the association between being exposed to offensive behaviors at work and WLE among older workers.

Offensive behaviors at work may include workplace bullying, defined as repeated and prolonged exposure to negative social acts involving a power imbalance between the parties concerned (13), and workplace violence or threats thereof, defined as isolated or repeated unacceptable behaviors/practices that may cause physical, psychological, sexual, or economic harm (14). While not the primary focus of this study, workplace bullying and violence may also entail expressions of discriminatory treatment (15), such as ageism. Meta-analytic evidence indicates that about 16% of employees globally experience workplace bullying and approximately 19% report exposure to physical violence at work (16). Offensive behaviors are increasingly recognized as social stressors at work and have been linked to adverse health outcomes, such depression and other common mental disorders, and the onset of cardiovascular disease (6, 17–21). In addition, exposure to offensive workplace behaviors has been linked to lower job satisfaction, increased intention to leave the job, and employee turnover or job change, suggesting a broader influence on employees’ working lives (22–26). Indeed, workplace bullying and workplace violence have been associated with a higher risk of temporarily or permanently exiting the labor market, manifested through sickness absence (27–32), unemployment (24), and disability pension (33, 34), although mixed findings have been reported for the latter (35). Several mechanisms have been proposed to underlie these associations, including stress (36, 37), poor health (31), and workplace expulsion or voluntary departure (24, 25). Overall, prior research on labor market exits after exposure to offensive behaviors at work has primarily examined specific exit routes, while no existing study has focused on quantifying these associations among older workers approaching the normative age of retirement.

Older workers may be particularly vulnerable to the labor market consequences of workplace offensive behaviors for several reasons. If these behaviors are driven by ageism, the psychological impact of violence and bullying at work may be compounded by the fact that they constitute an attack on one’s identity and self-worth. Empirical evidence further suggests that older workers may be more fragile to injuries following exposure to physical violence at work, potentially influencing their future work status (38). Additionally, older workers may have fewer opportunities for alternative employment, potentially prolonging exposure to adverse work environments, which may amplify associated health outcomes. Moreover, in Sweden, older workers, have a way to exit the labor market into old-age pension, something not possible to the same extent for younger workers.

This study sought to address this gap by examining whether exposure to workplace bullying and workplace violence or threats of violence were associated with shorter WLE, defined as the expected average number of working years, from a given age, that a person will spend in paid work, hereafter referred to as “work” (39, 40). As such, WLE can capture the dynamic nature of today’s labor market exits, often involving multiple transitions between being in and out of work, moving into part-time work, or winding down one’s long-term career before permanent exits. Specifically, our research objective was to quantify the associations between exposure to workplace bullying and workplace violence/threats of violence on the expected remaining years in working life from age 50.

## Methods

### Data material and study design

For this prospective cohort study, we used data from the Swedish Longitudinal Occupational Survey of Health (SLOSH), an ongoing cohort study initiated in 2006. Detailed information regarding SLOSH is available in the published cohort profile (41) and at www.slosh.se. In short, the first SLOSH wave (2006) was designed as a follow-up of the cross-sectional Swedish Work Environment Study (SWES), which was based on participants from the Swedish Labor Force Survey (stratified by work status). Since 2006, participants in SLOSH have been followed biennially (with annual follow-up starting 2022) through a questionnaire containing questions on a range of topics related to one’s current work, health, family, and labor market situation. SLOSH is also linked to the Swedish Longitudinal Integrated Database for Health Insurance and Labour Market Studies (LISA). SLOSH is an open cohort; nine refresher samples from SWES have been added to SLOSH since 2008. In 2024, the total SLOSH cohort consisted of 57 104 participants. Prior research found that both initial and repeated participation in SLOSH is more likely among women, older individuals, and individuals with higher educational attainment (41).

**Figure 1:**
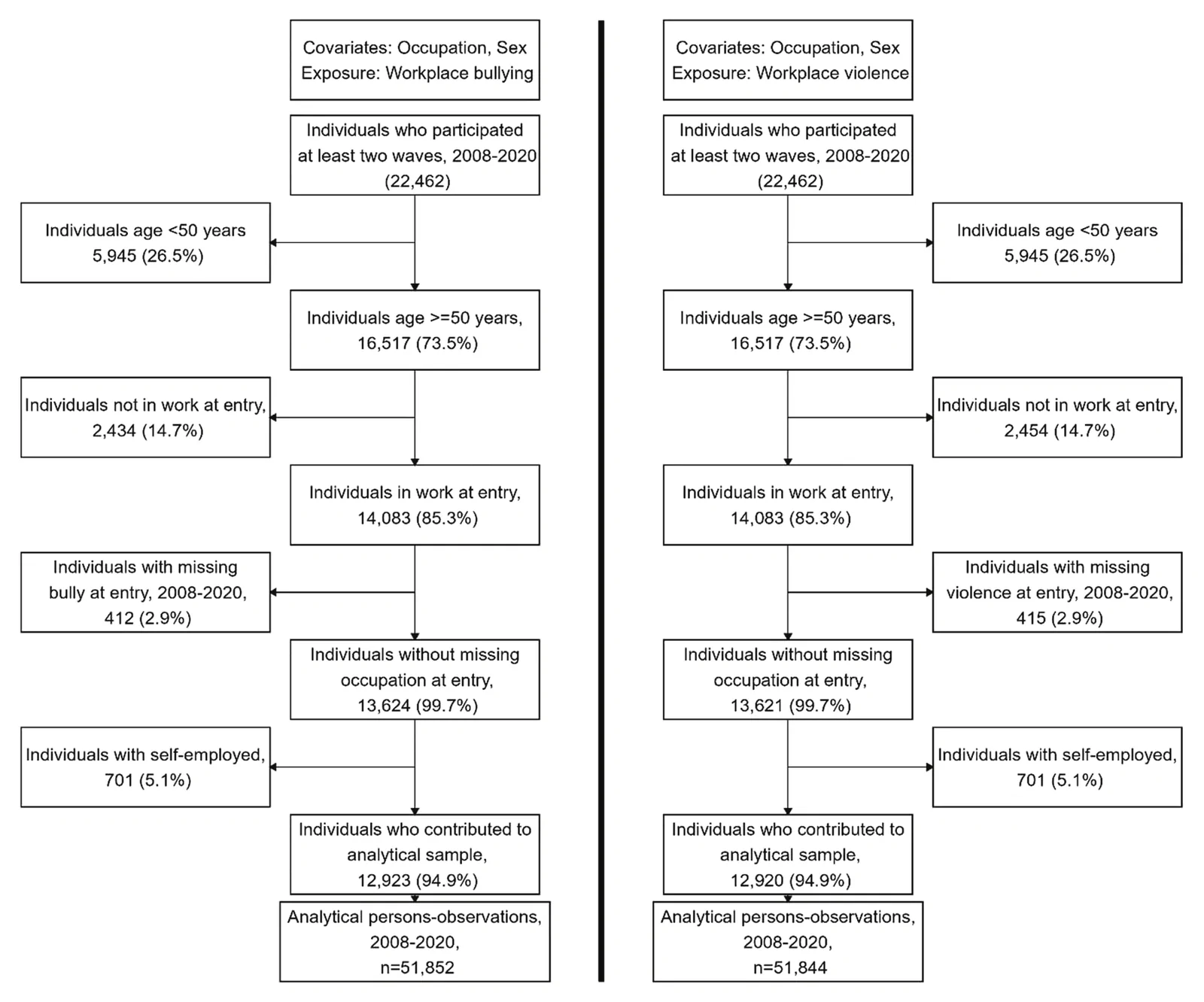
Flowchart of inclusion and exclusion of individuals and persons-observations included in the study.

**Figure 2:**
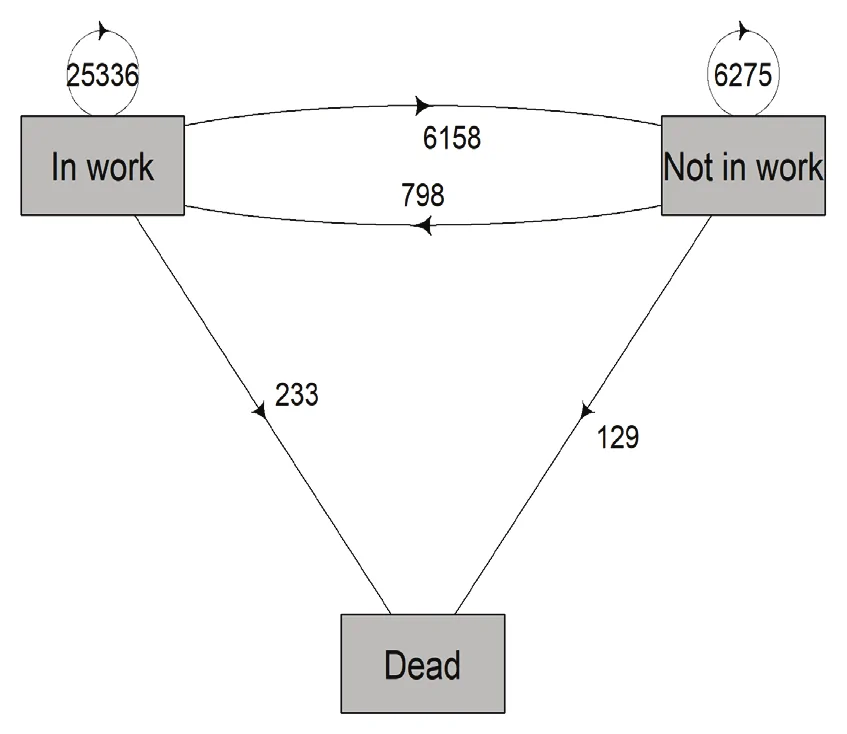
The observed number of transitions between and within the non-absorbing states (“in work” and “not in work”), as well as from the non-absorbing states to death, according to the three-state model to estimate working life expectancy.

In this study, we used data from the SLOSH surveys conducted in 2008 through 2020. Participants were eligible for inclusion if they took part in at least two survey waves during this period (though not necessarily in consecutive survey waves). To qualify, individuals had to be aged ≥50 and report being in paid work at the time of their first participation within this timeframe. Participants with missing information on work status, occupational level, and exposure to workplace bullying and/or workplace violence were excluded, as were self-employed individuals at baseline (given that self-employed individuals more often lack colleagues and supervisors, which limits the assessment of workplace bullying). Each participant’s baseline year was defined as the first year they met these criteria, meaning the baseline year could differ across individuals (2008, 2010, 2012, 2014, or 2018). The length of follow-up varied between individuals (range 2–12 years). Two analytical samples were created for the analyses of bullying (N=12 923) and violence/threats of violence (N=12 920), respectively (figure 1). Because the samples were largely identical, demographic and transitional characteristics are presented only for the larger sample (table 1, figure 2).

**Table 1:** Distributions of demographic characteristics, work status, and workplace offensive behaviors for individuals at entry (SLOSH, 2008–2018). [SD=standard deviation.]

Covariates		Men (N=5650)			Women (N=7273)	
		N (%)	Mean (SD)		N (%)	Mean (SD)
Age			56.6 (5.1)			56.0 (4.9)
Work status						
	Full-time	5271 (93.3)			6378 (87.7)	
	Part-time	379 (6.7)			895 (12.3)	
Occupational level						
	Professional	2170 (38.4)			2087 (28.7)	
	Intermediate	2074 (36.7)			3818 (52.5)	
	Routine	1407 (24.9)			1375 (18.9)	
Workplace bullying						
	Unexposed	5232 (92.6)			6488 (89.2)	
	Exposed	418 (7.4)			785 (10.8)	
Workplace violence						
	Unexposed	5232 (92.6)			6488 (89.2)	
	Exposed	418 (7.4)			785 (10.8)	

### Measurements

*Workplace bullying and workplace violence/threats of violence.* Exposure to workplace bullying and workplace violence/threats of violence were assessed via self-reports at baseline. Respondents were asked to indicate to what extent they had been exposed to “personal persecution in the form of unkind words or behaviors from superiors or fellow workers” (indicating bullying) and to “violence or threats of violence in your work”, respectively. Response options and time frame for exposure varied between survey years (supplementary material, www.sjweh.fi/article/4320 table S1). For both bullying and violence, the items were analyzed dichotomously with respondents reporting affirmative answers considered as exposed, and respondents reporting “no” or “not at all” considered unexposed. Cohen’s kappa estimates (supplementary table S2) suggest low agreement between the measures of workplace bullying and violence/threats of violence across survey waves (κ=0.08–0.11).

*Working life expectancy.* WLE was measured using accumulated data on an individual’s work and death statuses over time. Two recurrent states (in work, not in work) and one absorbing state (death) were included in the model. The recurrent states were based on self-reported data in SLOSH. We defined “in work” as working ≥10 hours per week and “not in work” as working <10 hours per week. The threshold of 10 hours per week reflects an arbitrary but pragmatic cut-off for a substantial contribution to working life, in line with our previous conceptualization of WLE (12). Information on age at death was retrieved from the Cause of Death Register kept by the Swedish Board of Health and Welfare.

*Covariates.* Information on age (in years) and sex (female/male) was retrieved from the LISA-register. Occupational level was measured using self-reported job title and coded according to the Swedish socioeconomic classification. Following previous research (12), we collapsed occupational level into three groups: routine (eg, manual workers), intermediate (eg, assistant, and intermediate non-manual employees), and professional (eg, executives, professionals, and other high level non-manual employees).

### Analytical approach

We applied multi-state survival models to estimate transition probabilities between different statuses (in work, not in work) and death (the absorbing state). These models incorporated stochastic processes to account for dynamic labor market trends and mortality patterns influencing older workers’ trajectories. Unlike prevalence-based methods (eg, the Sullivan method) (31), multi-state models permit bidirectional transitions (eg, re-entry into work after not being in work), enabling robust estimates of marginal working life expectancies that reflect individuals’ cumulative exposure to labor market risks over time.

Hazard rates for state transitions were modeled using the *msm* package (32) in *R* (33), with age as a continuous covariate under a Gompertz function. This approach assumes log-linear, piecewise-constant transition probabilities within defined age intervals (34). To compute working life expectancies, we employed the *elect* package (34), which extends the msm framework by integrating Gompertz-based hazard extrapolation. The analyses were first conducted in the total study sample and subsequently in stratified subsamples defined by sex, occupational level, and the combined categories of sex and occupational level. Both absolute WLE estimates and differences in WLE between exposed and unexposed participants were calculated, together with 95% confidence intervals (CI). The CI were derived from 500 parametric bootstrap simulations, leveraging asymptotic properties of maximum likelihood estimators (34, 35). To obtain an overall estimate that corresponds to a model adjusted for sex and occupational level, we pooled the sex- and occupation-specific estimates using fixed-effect meta-regression with inverse-variance weighting and calculated the adjusted difference in WLE between exposed and unexposed participants.

The exact timing of transitions between non-absorbing states (eg, in work to not in work) between two survey waves are not known; thus, the non-absorbing states were treated as interval-censored. The *msm* package’s censoring facility accommodated this uncertainty, ensuring unbiased parameter estimates despite unobserved exact transition times.

## Results

Table 1 shows the distribution of demographic characteristics, work status, and exposure to workplace offensive behaviors for the study sample at their given baseline year (SLOSH 2008–2018), stratified by sex. A larger proportion of women were in part-time work at baseline. A larger proportion of men than women had routine or professional level occupations, whereas women clustered in intermediate occupations. Among women, 10.8% reported exposure to workplace bullying and 10.8% reported exposure to workplace violence/threats of violence. Among men, that proportion was 7.4% for bullying and 7.4% for violence/threats of violence.

Figure 2 shows the observed number of transitions between the three states (in work, not in work, dead) derived from the three-state Markov models. During the study period, there were 6158 transitions from “in work” to “not in work”, and 798 transitions from “not in work” to “in work”. Transitions were further observed from both “in work” and “not in work” to death (233 and 129 transitions, respectively). A large majority (25 336) remained in the “in-work” state during the entire study period. Based on these models, we proceeded to estimate total WLE from age 50.

Table 2 shows the estimated WLE, including 95% CI, for individuals exposed and unexposed to workplace bullying. In the total sample, the estimated WLE from age 50 was shorter (-0.42 years, 95% CI -1.23–0.01) for individuals having experienced exposure to workplace bullying, compared to unexposed individuals. Analyses stratified by sex, by occupational level, and their combination (supplementary table S3) showed consistent patterns, with lower WLE among bullied compared to not bullied participants across all strata, with statistically significant differences observed among individuals in intermediate occupations. After adjusting for sex and occupational level, exposure to workplace bullying was associated with a -0.63-year difference in WLE (95% CI -1.39–0.14) compared to those unexposed. However, this difference was not statistically significant (table 2, supplementary table S4).

**Table 2:** Estimated total working-life expectancies (WLE) at age 50 for individuals exposed and not exposed to workplace bullying, and the corresponding crude and adjusted differences in WLE. [SE=standard error; CI=confidence interval.]

		WLE			Difference in WLE (95% CI)	Adjusted difference in WLE (95% CI) ^a^
		Estimate	SE	95% CI		
All						
	Unexposed	12.91	0.07	12.77–13.03		
	Exposed	12.49	0.36	11.64–12.84	-0.42 (-1.23–0.01)	-0.63 (-1.39–0.14)
Men						
	Unexposed	13.17	0.10	12.95–13.35		
	Exposed	12.80	0.62	11.46–13.20	-0.37 (-1.67–0.07)	
Women						
	Unexposed	12.69	0.09	12.49–12.84		
	Exposed	12.32	0.34	11.63–12.65	-0.36 (-1.00–0.00)	
Professional						
	Unexposed	13.55	0.14	13.28–13.81		
	Exposed	13.11	0.55	11.91–13.53	-0.44 (-1.63–0.05)	
Intermediate						
	Unexposed	12.79	0.10	12.58–12.96		
	Exposed	12.35	0.54	11.41–12.73	-0.44 (-1.37– -0.01)	
Routine						
	Unexposed	12.66	0.12	12.37–12.87		
	Exposed	12.27	0.53	11.15–12.66	-0.39 (-1.42–0.07)	

^a^ Adjusted for sex and occupational level. Estimate derived from fixed-effect meta-regression with inverse-variance weighting.

Table 3 shows WLE, including 95% CI for individuals exposed and unexposed to workplace violence/threats of violence. In the total sample, the estimated WLE from age 50 was shorter (-0.42 years, 95% CI -1.06–0.01) for individuals having experienced exposure to workplace violence/threats of violence, compared to unexposed individuals. Again, a similar pattern was observed across all strata of sex and of occupational level, as well as their combination (supplementary table S5), although these differences in WLE were not statistically significant. In the analysis adjusted for sex and occupational level, we found a statistically significant difference in WLE between exposed and unexposed individuals. Specifically, exposure to violence/threats of violence was associated with a -0.56-year difference in WLE (95% CI -1.02– -0.10) compared to those unexposed (i.e., approximately 6.7 months) (table 2, supplementary table S6).

**Table 3:** Estimated total working-life expectancies (WLE) at age 50 for individuals exposed and not exposed to workplace violence, and the corresponding crude and adjusted differences in WLE. [SE=standard error; CI=confidence interval].

		WLE			Difference in WLE (95% CI)	Adjusted difference in WLE (95% CI) ^a^
		Estimate	SE	95% CI		
All						
	Unexposed	12.91	0.06	12.77–13.02		
	Exposed	12.49	0.26	11.85–12.87	-0.42 (-1.06–0.01)	-0.56 (-1.02– -0.10)
Men						
	Unexposed	13.17	0.10	12.97–13.35		
	Exposed	12.81	0.58	11.52–13.21	-0.36 (-1.66–0.09)	
Women						
	Unexposed	12.69	0.09	12.49–12.84		
	Exposed	12.33	0.62	11.51–12.68	-0.36 (-1.16–0.07)	
Professional						
	Unexposed	13.55	0.13	13.28–13.77		
	Exposed	13.12	0.63	12.31–13.54	-0.43 (-1.22–0.05)	
Intermediate						
	Unexposed	12.79	0.10	12.55–12.93		
	Exposed	12.36	0.32	11.77–12.78	-0.43 (-1.06–0.05)	
Routine						
	Unexposed	12.66	0.12	12.38–12.88		
	Exposed	12.28	0.71	10.51–12.68	-0.38 (-2.28–0.10)	

^a^ Adjusted for sex and occupational level. Estimate derived from fixed-effect meta-regression with inverse-variance weighting.

## Discussion

In this population-based prospective Swedish cohort study, exposure to workplace violence/threats of violence was associated with a shorter WLE among workers from age 50. This association was statistically significant after adjusting for sex and occupational level, with the adjusted difference estimated at 0.56 years, equivalent to approximately 6.7 months per exposed individual. Exposure to workplace bullying was also associated with a shorter WLE, although this difference was not statistically significant when adjusting for sex and occupational level. Together, these findings suggest that offensive behaviors at work may influence the duration of working lives among older workers.

To the best of our knowledge, this is the first study examining associations between offensive behaviors at work and WLE. Our results are in line with previous studies that have reported associations between both exposure to workplace bullying and exposure to violence/threats of violence and subsequent temporary and permanent labor market exits, including sickness absence (27–32), unemployment (24), and disability pension (33, 34), though direct comparisons are complicated by differences in outcome operationalizations. The results from our study are also in line with previous research from Scandinavian samples showing that poor psychosocial working conditions are associated with shorter WLE among workers at age 50 (10, 42, 43). The magnitude of the difference observed in our study is comparable to that reported for high versus low effort-reward imbalance in a Finnish sample of public sector employees (approximately 5 months), and somewhat smaller than estimates reported for high versus low job strain (6–12 months) (12) and low job control (approximately one year) (42) in samples based on the general Swedish workforce.

The -0.56-year difference in WLE at age 50 between individuals exposed to workplace violence and those unexposed can be translated into individual-level costs, such as lower pension accrual and higher reliance on sickness absence or disability benefits. According to the Swedish Pension Agency, working one year less before retirement would result in approximately 1900– 2300 SEK less in pension per month (44). Previous studies have reported a prevalence of 11–17% of workplace violence or threats of violence in the general working population in Sweden with a mean age of around 50 years (45). Therefore, this -0.56-year difference is likely to translate into a substantially larger impact at the population level, especially concerning lost tax revenue, added benefit costs, and reduced pension capital.

### Implications

The present study found that exposure to offensive behaviors at work is associated with shorter remaining WLE at age 50. At the individual level, the findings highlight that outcomes of exposure to offensive behaviors at work may extend beyond immediate changes in health to influence long-term labor market participation, potentially influencing financial security in retirement. For employers, the findings underscore the need for primary prevention of offensive behaviors at work. This may be particularly relevant in certain sectors, such as healthcare, where exposure to violence is highly prevalent (46) and workforce retention is already a considerable challenge, although more research is needed to examine sector-specific differences in WLE. Finally, our findings are relevant in the context of ongoing efforts to extend working lives in aging populations (3). If offensive behaviors at work contribute to shorter WLE, addressing these behaviors may be one avenue for supporting longer labor market participation among older workers. Given that WLE-estimates express associations in years, it may be a useful measure that is easy to interpret when communicating the potential labor market relevance of poor working conditions to policy-makers.

### Strengths and limitations

This is the first study to estimate WLE in relation to exposure to offensive behaviors at work. One of the key advantages of this study is the use of a large and longitudinal dataset. By employing multi-state modeling techniques instead of relying on more traditional metrics such as the average retirement age, this study better captures the dynamic nature of current labor-market exits. These models also allowed us to account for deaths, a competing transition, which is likely to reduce bias in estimates of length of working life.

The results of the study should be considered in light of several limitations. First, our measure of WLE relied on self-reported information on the number of working hours, where individuals who worked <10 but >0 hours were also treated as “not in work”. Our data did not allow us to distinguish between different reasons for not being in work (eg, long-term sickness absence, permanent disability pension, unemployment). Moreover, limited sample size prevented us from distinguishing between working full-time and working part-time in our multi-state model, thus introducing a risk of misclassification. For instance, individuals who transitioned from full-time work to part-time work (eg, due to part-time sickness absence or partial disability pension or old-age retirement), were still classified as remaining in the in-work state provided that their total working time exceeded 10 hours per week. If such transitions occurred more frequently among exposed individuals, our estimates may underestimate the impact of exposure to offensive behaviors on WLE. Limited sample size also meant that relatively few of the individuals included in our analyses were categorized as exposed, which may have reduced statistical power needed to detect associations.

Second, due to the statistical complexity of multi-state modelling, we could not adjust our analysis for any other covariates potentially related to both WLE and offensive behaviors, beyond sex and occupational level. In our case, factors such as birth country or baseline mental health may have influenced both the likelihood of (reporting) exposure to workplace offensive behaviors as well as the risk of early labor market exit. Furthermore, as workplace violence exposure may be occupation-specific, likely concentrated in roles facing external parties such as clients, patients, or pupils, the broad occupational level categorization used here may not fully account for within-group variation in violence risk, and residual confounding by occupation cannot be excluded. This may have contributed to an overestimation of the association between workplace violence and reduced WLE.

Third, we only measured exposure at one point in time. Some misclassification might thus have occurred as individuals classified as exposed at baseline may have experienced exposure to bullying or violence at a later time point. Moreover, whether baseline exposure reflected accumulated or isolated episodes of bullying/violence is unknown. Because exposure could only be measured while participants were working, some misclassification may have occurred near employment exit, potentially leading to underestimation of the association with WLE. The perpetrator of the offensive acts was also not fully identified: bullying was limited to colleagues or supervisors, whereas violence could involve individuals both inside and outside the organization, eg, patients or clients. The lack of differentiation between perpetrators of violence may also have introduced some overlap with the bullying measure. However, kappa values assessing agreement between the two measures were low, lending confidence to the interpretation of their associations with WLE as reflecting partly distinct phenomena. Moreover, the measures of bullying and violence were based on self-reports, using single items without definitions of the concepts. Misclassification due to underreporting or failure to capture exposure to certain behaviors associated with bullying or violence might have contributed to bias towards the null.

Fourth, selection might have influenced the results. Highly educated individuals and individuals born in Sweden, generally less affected by workplace bullying and violence, are for example overrepresented in the SLOSH sample. Prior research has also indicated that individuals who participate in multiple SLOSH-waves are healthier than those who only take part once (41), potentially leading to an underestimation of the true impact on WLE.

Lastly, this study aimed at estimating WLE for older workers after exposure to workplace offensive behaviors, and thus, we cannot determine how workplace offensive behaviors might alter the working lives of younger workers, under the age of 50. The potential reduction in WLE could be considerably greater if exposure leads to premature labor market exit at a younger age, including permanent exits. We, therefore, recommend that future studies examine these associations also in younger populations.

### Concluding remarks

Our analysis shows that older employees who have been exposed to violence or threats of violence in the workplace, on average, can be expected to have a shorter working life from the age of 50. This corresponds to approximately six months less of WLE than among those who have not experienced such violence. Given the number of older workers potentially affected by these types of offensive behaviors at work, this reduction could have costly societal and economic implications, not the least in the context of ongoing demographic changes. The findings from this study, thus, underscore the potential importance of preventive interventions targeting workplace violence, although further research is needed to confirm these associations and to evaluate effective intervention strategies.

## Supplementary material

Supplementary material

## Protection of research participants

Thus study was conducted in accordance with the Helsinki Declaration as revised in 2008. Ethical approval for SLOSH has been obtained from the Regional Ethical Review Board in Stockholm (#2012/373‐31/5, #2006/158‐31, #2008/240‐32, #2008/1808‐32, #2010/0145‐32, #2012/373‐31/5, #2013/2173‐32, #20152187, #2015/2298‐32, #2017/25‐35‐32). Participants in SLOSH are informed about the purpose of the survey, that their participation is voluntary, and that they have the right to withdraw from participation at any time.
