# Supplementary material for: Exposure to workplace bullying and workplace violence/threats of violence and subsequent working life expectancy among older workers: A Swedish prospective cohort study
Source: Scand J Work Environ Health. 2026 Aug 31;52(5):560–9. doi: 10.5271/sjweh.4320 (PMC13551369; doi:10.5271/sjweh.4320)
Supplement: Supplementary file 1 [file SJWEH-52-560-S001.pdf]

Exposure to workplace bullying and workplace violence/threats of violence and subsequent working life expectancy among older workers: A Swedish prospective cohort study[1]

by Rebecca Holmgren, PhD, [2] Holendro Singh Chungkham, PhD, Linda L Magnusson Hanson, PhD, Tianwei Xu, PhD, Kristina Alexanderson, PhD, Kristin Farrants, PhD, Martin Hyde, PhD, Robin S Högnäs, PhD, Reiner Rugulies, PhD, Hugo Westerlund, PhD

1. Supplementary material
2. Correspondence to: Rebecca Holmgren, PhD, Division of Psychobiology and Epidemiology, Department of Psychology, Stockholm University, Albanovägen 12, 114 19 Stockholm, Sweden. [E-mail: rebecka.holmgren@su.se]

Table S1**. Items measuring exposure to workplace bullying and workplace violence/threats of violence in SLOSH, by baseline survey year.**

Survey year	Workplace bullying	Workplace violence/ threats of violence	Response options
2008	Are you subjected to personal persecution in the form of unkind words or behaviors from your superiors or fellow workers?	Are you subjected to violence or threats of violence in your work?	Every day<br>A couple of days a week (1 day of 2)<br>One day a week (1 day of 5)<br>A couple of days a month (1 day of 10)<br>Sometimes during the last 3 months<br>Once or twice during the last 12 months<br>Not at all during the last 12 months
2010	Are you subjected to personal persecution in the form of unkind words or behaviors from your superiors or fellow workers?	During the last 12 months, have you been subjected to violence or threats of violence in your work?	Yes<br>No
2012, 2014, 2016, 2018	During the last 6 months, have you been subjected to personal persecution in the form of unkind words or behaviors from your superiors or fellow workers?	During the last 6 months, have you been subjected to violence or threats of violence in your work?	Weekly<br>Monthly<br>Sometimes<br>No

Table S2. **Cohen's kappa statistics for agreement between measures of workplace bullying and workplace violence in SLOSH**

SLOSH year	Cohens kappa (95% CI)
2008	0.08 (0.04─ 0.12)
2010	0.08 (0.04─ 0.12)
2012	0.10 (0.06─ 0.14)
2014	0.08 (0.05─ 0.11)
2016	0.09 (0.06─ 0.12)
2018	0.09 (0.06─ 0.13)
2020	0.11 (0.07─ 0.14)
All waves[a]	0.21 (0.15─ 0.26)

[a]Modified Kappa, accounting for the repeated nature

Table S3: **Estimated total working life expectancies (WLE) and differences in WLE at age 50 by workplace bullying exposure status, occupational level, and sex.**

	Working life expectancy (WLE)			Difference in WLE (95% CI)
**Covariates/exposure**	**Estimate**	**SE[a]**	**95%CI[b]**	
**Men**				
**Professional**				
Unexposed	13.76	0.16	13.45— 14.09	
Exposed	13.30	1.59	10.19— 13.81	-0.46 (-2.04— 0.16)
**Intermediate**				
Unexposed	13.07	0.13	12.81— 13.33	
Exposed	12.63	1.29	10.10— 13.09	-0.44 (-1.85— 0.07)
**Routine**				
Unexposed	12.84	0.15	12.55— 13.11	
Exposed	12.43	1.33	9.81— 12.96	-0.41 (-3.00— 0.22)
**Women**				
**Professional**				
Unexposed	13.25	0.16	12.95— 13.55	
Exposed	12.86	0.89	11.11— 13.34	-0.39 (-2.51— 0.18)
**Intermediate**				
Unexposed	12.60	0.10	12.40— 12.80	
Exposed	12.21	0.52	11.18— 12.60	-0.39 (-1.86— 0.07)
**Routine**				
Unexposed	12.39	0.14	12.11— 12.65	
Exposed	12.02	1.95	8.21— 12.51	-0.37 (-3.24— 0.20)

[a]SE: Standard error. [b] CI: Confidence interval.

Table S4: **Results from fixed-effect meta-regression showing adjusted differences in working-life expectancy (WLE) at age 50 by workplace bullying, sex, and occupational level.**

**Variables in the meta-regression**	**Difference in WLE (95%CI)**
Workplace bullying (yes vs no)	-0.63 (-1.39─ 0.14)
Sex (women vs men)	-0.48 (-0.69─ -0.26)
Occupational level (routine vs professional)	-0.65 (-0.91─ -0.38)
Occupational level (intermediate vs professional)	-0.84 (-1.14─ -0.55)
Constant[a]	13.72 (13.47─ 13.97)

[a]The constant represents the estimated WLE at age 50 for the reference group.

Table S5: **Estimated total working life expectancies (WLE) and differences in WLE at age 50 by workplace violence exposure status, occupational level, and sex.**

	Working life expectancy (WLE)			Difference in WLE (95% CI)
**Covariates/exposure**	**Estimate**	**SE**[a]	**95%CI**[b]	
**Men**				
**Professional**				
Unexposed	13.75	0.15	13.46— 14.04	
Exposed	13.29	0.68	11.95— 13.82	-0.46 (-1.84— 0.19)
**Intermediate**				
Unexposed	13.06	0.15	12.77— 13.29	
Exposed	12.61	0.66	11.32— 13.10	-0.45 (-1.59— 0.11)
**Routine**				
Unexposed	12.82	0.15	12.53— 13.09	
Exposed	12.48	1.23	10.07— 12.97	-0.34 (-1.86— 0.14)
**Women**				
**Professional**				
Unexposed	13.27	0.16	12.96— 13.54	
Exposed	12.84	0.53	11.80— 13.32	-0.43 (-1.88— 0.10)
**Intermediate**				
Unexposed	12.60	0.10	12.40— 12.80	
Exposed	12.21	0.32	11.59— 12.63	-0.39 (-1.17— 0.06)
**Routine**				
Unexposed	12.41	0.14	12.13— 12.69	
Exposed	12.03	0.77	10.53— 12.51	-0.38 (-2.28— 0.19)

[a] SE: Standard error. [b] CI: Confidence interval.

Table S6: **Results from fixed-effect meta-regression showing adjusted differences in working-life expectancy (WLE) at age 50 by workplace violence/threats of violence, sex, and occupational level.**

**Variables in the meta-regression**	**Difference in WLE (95%CI)**
Workplace violence/threats (yes vs no)	-0.56 (-1.02─ -0.10)
Sex (women vs men)	-0.47 (-0.69─ -0.25)
Occupational level (routine vs professional)	-0.61 (-0.88─ -0.35)
Occupational level (intermediate vs professional)	-0.77 (-1.05─ -0.49)
Constant[a]	13.68 (13.44─ 13.91)

[a]The constant represents the estimated WLE at age 50 for the reference group.
